# Ontology-Based Meta-Analysis of Global Collections of High-Throughput Public Data

**DOI:** 10.1371/journal.pone.0013066

**Published:** 2010-09-29

**Authors:** Ilya Kupershmidt, Qiaojuan Jane Su, Anoop Grewal, Suman Sundaresh, Inbal Halperin, James Flynn, Mamatha Shekar, Helen Wang, Jenny Park, Wenwu Cui, Gregory D. Wall, Robert Wisotzkey, Satnam Alag, Saeid Akhtari, Mostafa Ronaghi

**Affiliations:** 1 NextBio, Cupertino, California, United States of America; 2 Royal Institute of Technology (KTH), Stockholm, Sweden; 3 Illumina, San Diego, California, United States of America; Cairo University, Egypt

## Abstract

**Background:**

The investigation of the interconnections between the molecular and genetic events that govern biological systems is essential if we are to understand the development of disease and design effective novel treatments. Microarray and next-generation sequencing technologies have the potential to provide this information. However, taking full advantage of these approaches requires that biological connections be made across large quantities of highly heterogeneous genomic datasets. Leveraging the increasingly huge quantities of genomic data in the public domain is fast becoming one of the key challenges in the research community today.

**Methodology/Results:**

We have developed a novel data mining framework that enables researchers to use this growing collection of public high-throughput data to investigate any set of genes or proteins. The connectivity between molecular states across thousands of heterogeneous datasets from microarrays and other genomic platforms is determined through a combination of rank-based enrichment statistics, meta-analyses, and biomedical ontologies. We address data quality concerns through dataset replication and meta-analysis and ensure that the majority of the findings are derived using multiple lines of evidence. As an example of our strategy and the utility of this framework, we apply our data mining approach to explore the biology of brown fat within the context of the thousands of publicly available gene expression datasets.

**Conclusions:**

Our work presents a practical strategy for organizing, mining, and correlating global collections of large-scale genomic data to explore normal and disease biology. Using a hypothesis-free approach, we demonstrate how a data-driven analysis across very large collections of genomic data can reveal novel discoveries and evidence to support existing hypothesis.

## Introduction

High-throughput technologies have become essential tools for biological researchers. The advent of “open biology” has led to an exponential growth of high-throughput data in publicly shared repositories, such as NCBI GEO, EBI Array Express, and the Stanford Microarray Database (SMD) [1]. The billions of data points collected within these repositories provide an unprecedented opportunity for exploring and comparing molecular portraits of different biological states. However, the complex and heterogeneous nature of this exponentially growing amount of data has created a new and daunting challenge for a community wishing to explore it in a systematic and easy way.

A number of meta-analysis studies across multiple sets of gene expression data have led to important discoveries, such as: i) the identification of consistently and significantly deregulated genes in prostate cancer [2], ii) the derivation of candidate biological pathways that underlie mechanisms of carcinogenesis [3], and iii) the identification of lung adenocarcinoma genetic markers that correlated with patient survival [4], among others [5]–[10]. These studies typically focused on a single phenotype and identified significant differentially expressed sets of genes across multiple datasets. Conversely, an investigator-generated gene signature can be applied across large collections of high-throughput data to look for associations with various diseases, tissues, and treatments. In the landmark study by *Lamb et al.*, the connections between disease-associated gene expression “footprints” and gene expression profiles of different cell lines treated with diverse compounds were explored through meta-analysis of data generated on a highly standardized, single microarray platform [11]. The authors created a map that linked disease to relevant compounds by computing enrichment-based “connectivity” scores between their corresponding gene expression signatures. Public repositories, however, contain thousands of independent studies with highly heterogeneous data from different labs, platforms, and organisms. The high level of complexity makes it difficult to use by the broader scientific community.

Here we report the development of a novel strategy to explore the biological properties of gene sets found in global collections of public or proprietary large-scale experimental data. The size of gene sets queried can range from the tens (e.g., the results of qPCR experiments) to hundreds or even thousands (e.g., the gene signature results from microarray or next generation sequencing experiments). Using a unique combination of rank-based enrichment algorithms, ontologies, and meta-analysis techniques, we compute correlation scores between a given gene set and thousands of public studies. The output provides a ranked set of signatures and “meta-concepts” representing diseases, normal tissues, compound treatments, and genetic perturbations (gene mutations, knockouts, siRNA knockdowns) that have strong association with a gene set of interest. We applied our strategy to develop NextBio (www.nextbio.com) – a data mining framework that integrates and correlates global public datasets with the user's own experimental data. As a demonstration, we used NextBio to detect and explore the biology of brown fat, to compare its expression profile to those of other tissues and cell types, and to discover connectivities with different disease states and chemical and genetic perturbations.

## Results

### Data pre-processing and correlation overview

**Figure 1:**
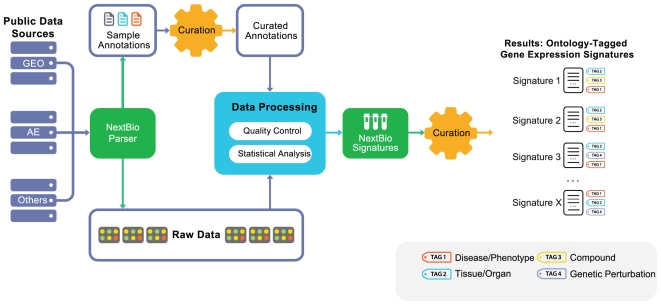
Public data processing and analysis pipeline diagram. The steps for turning public datasets into processed gene signatures include: raw data collection, sample annotation curation, data quality control, automated analysis, and manual tagging of resulting signatures with disease, tissue, compound ontology, and gene perturbation terms (tags). Curation of sample annotation includes a systematic analysis of all sample attributes that should be processed for differential expression. The data processing step converts original raw data into processed results – gene expression signatures representative of a given biological condition. The final tagging step ensures that key biological conditions associated with each signature are captured with standardized vocabulary terms, enabling downstream meta-analysis.

**Figure 2:**
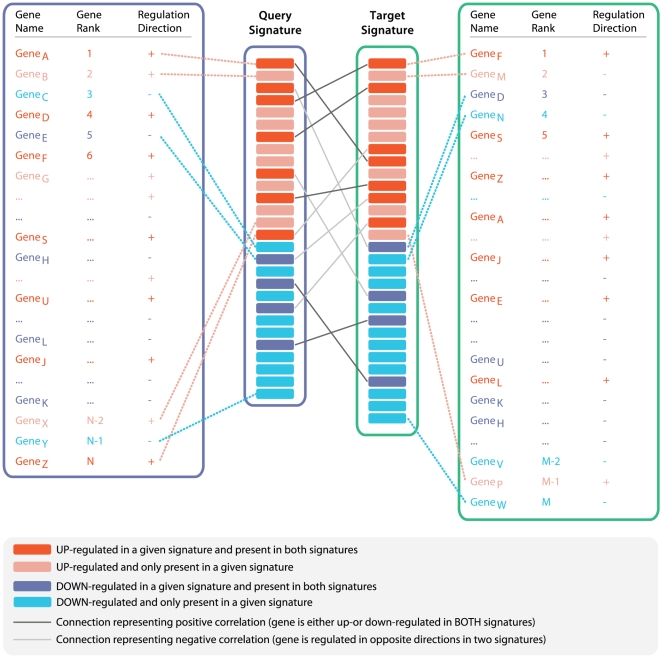
Computing pairwise signature correlation scores. The algorithm represented by this schematic computes an enrichment score and p-value between two ranked gene signatures. Dark red and blue colored boxes indicate genes present in both signatures; light red and blue colored boxes represent genes present in only one of the signatures. Dark lines connecting genes in each signature represent connections between genes with the same direction of regulation in both signatures. Light lines connect genes with opposite direction in two signatures.

**Figure 3:**
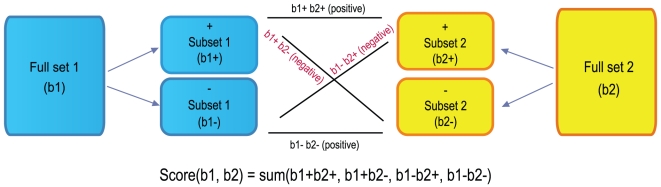
Computing directionality and final correlation scores between two signatures. The directional subsets are formed for both b1 and b2, and subset-subset enrichment scores are Computed for b1^+^b2^+^, b1^+^b2^−^, b1^−^b2^+^, and b1^−^b2^−^. Pairwise correlation scores for the directional subsets are positive where subsets are of the same direction and negative sign otherwise. The correlation scores of the subsets are summed up to give the final score for full set b1 versus full set b2.

**Figure 4:**
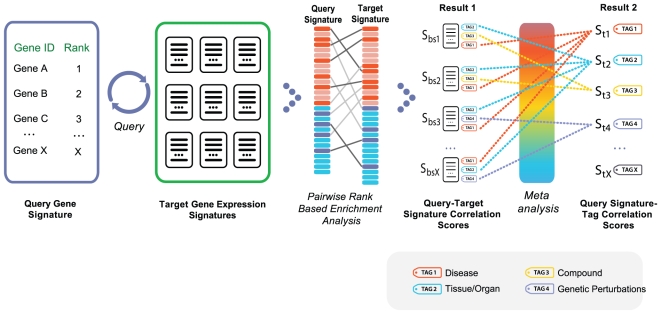
Gene signature query against all other signatures within the system. First, pairwise gene signature correlation scores (using rank-based enrichment statistics) are computed, followed by meta-analysis of individual score-tag pairs to compute overall tag scores. This two step process results in computation of direct correlations between user's defined signature and diverse biological conditions representing normal tissues and cell types, diseases, and compounds. Furthermore, overall positive or negative correlation between a signature and a concept is computed based on individual pairwise signature correlation scores. A positive correlation implies a similar up- and down-regulation of genes in each signature or signature-tag pair, while a negative correlation implies the opposite trend.

Our data mining strategy can be divided into two parts. In the first part (Figure 1), semi-automated crawlers collected public data from diverse sources, such as NCBI GEO [12], Array Express [13], SMD [14], Broad Cancer Genomics [15], Cancer Biomedical Informatics Grid (caBIG), and other repositories (Table S1). A data analysis step produced sets of differentially expressed gene signatures associated with each experimental or clinical comparison, such as disease versus normal (Methods). In the final step of part one, all signatures were tagged with relevant ontology terms (Figure 1) that reflected associated tissue types, disease/phenotype, compound treatment, or genetic perturbation (e.g., gene mutation, knockout, siRNA knockdown). In the second part, rank-based enrichment statistics were applied to compute pairwise correlation scores between all signatures (Figures 2 and 3) followed by a meta-analysis to compute individual signature-ontology concept correlation scores (Figure 4).

To ensure that data were comparable across different platforms and species, gene signature identifiers were translated using both a universal gene dictionary to map them to a standard NCBI gene reference and a cross-organism dictionary to assign them to precomputed ortholog clusters (Methods). Gene annotations and probe definitions were regularly revised to ensure that they are up-to-date with manufacturer specifications. While issues have been reported with microarray spot definitions [16]–[18], we believe that our meta-analysis approach mitigated the effects of errors that occur on a single platform since the strength of an association was weighted by its consistency across multiple studies and platforms.

We collected and analyzed over 6,000 individual experiments from different public sources of large-scale experimental data. Within this collection were more than 140,000 individual samples profiled on gene expression microarrays. Out of 6,000 experiments, only 4,000 passed our extensive quality control (QC) criteria. Approximately 60% of the disqualified studies were excluded from processing due to insufficient replicates, lack of control samples, or unsupported platforms (e.g., platforms that do not cover more than half the number of genes for an organism). Approximately 25% of the disqualified studies were duplicated (e.g. part of an already processed super-series), and 15% were excluded for failing QC metrics during pre-processing and differential expression analysis (Table 1, Methods).

**Table 1:** Summary of all data associated with normal tissues, diseases, drug treatments, and genetic perturbations.

Concept Type	Total Studies	Total Signatures	Total Samples	Total Concepts
Normal Tissues	450	2,120	14,500	120
Diseases	1,390	5,880	54,600	700
Compounds	990	10,830	45,420	1,430
Genetic Perturbations	*1,235*	*6,170*	*16,400*	135
**Total**	**4,065**	**25,000**	**130,920**	**2,375**

Total concepts count represents the number of specific ontology terms that are assigned as tags to signatures, or which represent “parent” ontology concepts. For example, when a gene signature is tagged with “heart ventricle”, it is automatically considered tagged with the parent term “heart” and both are considered in the counts shown. “Total studies” refers to the number of studies that contain gene signatures that contribute to a given concept based on their associated tags. “Total Signatures” and “Total Samples” refer to the number of gene signatures and individual samples contributing to a given concept type (e.g. disease).

After applying a statistical analysis to identify differentially expressed genes in each experiment, we obtained a total of 25,000 gene signatures (a typical study produces multiple results). Each signature was tagged with relevant ontology terms. This annotation step identified a total of 120 unique normal tissue concepts, 700 disease, 1,430 compound, and 135 genetic perturbation concepts (including gene mutations, knockouts, and siRNA knockdowns). The final dataset contained a high-dimensional space of gene signatures with ranked genes and associated ontology concepts (tags) for diseases, tissues, compounds, and genetic perturbations (Table 1).

### Computing pairwise signature correlation (enrichment) scores

A number of factors must be considered when performing a comparative analysis of highly heterogeneous data from different sources, platforms, and technologies. We applied statistical methods to compensate for differences in platforms and their probe content, in organisms studied, and in signature sizes that could arise from choices of analysis stringency (e.g. p-value cutoffs). In addition, directional information (up- or down-regulation) is important for assessing connectivity between different gene sets derived from gene expression data. Our rank-based directional enrichment analysis enabled us to statistically assess pairwise correlations between any two gene signatures and to use this information to rank connectivity between different biological states.

We applied our algorithm to compute pairwise correlation scores between all signatures in our system (Figure 2). The magnitude of the pairwise correlation score reflected the similarity of the two signatures, which is measured by the extent that the genes in one signature set are enriched at the top ranks of the other signature set, and vice versa. Each signature consisted of a list of genes that passed a select fold change, p-value, or other test statistic threshold. Thresholds might vary between different researchers, types of studies, and analytical methods, and thereby result in different signature sizes. To capture the key enrichment signal, even among very short or very long signatures, we developed a non-parametric rank-based statistical approach.

The general design of the algorithm, which we call “Running Fisher” (see Methods), is analogous to the Gene Set Enrichment Analysis (GSEA) method [11], [19]. As with GSEA, Running Fisher dynamically detects the most significant enrichment signal in a ranked signature, allowing the signature to contain a relatively more comprehensive collection of genes than would otherwise be required when using a stringent statistical cutoff. This “dynamic” enrichment detection approach overcomes the limitations of a more commonly used “selection” approach where a too stringent cutoff might lead to potential loss of significant information, and a too relaxed cutoff might include insignificant data into the evaluation [20]. The Running Fisher algorithm differs from GSEA in the assessment of the statistical significance, where p-values are computed by a Fisher's exact test rather than by permutations (see Methods for details). Overall, this approach provided us the flexibility to compute correlation scores for data of different sizes and filter thresholds, as well as the ability to use ranks in both query and target signatures.

The directional relationship between the two signatures was captured by the sign of the correlation score. The up-regulated genes and the down-regulated genes were separated into directional subsets, and correlation scores were computed for each directional subset from one signature against each subset from the other signature (Figure 3). A positive sign was given to a subset pair that changed expression in the same direction, and a negative sign was given to a subset pair that changed in opposite directions. The overall correlation score was the sum of directional subset scores, and the sign of the sum determined whether the two signatures were positively or negatively correlated (see Methods for details). Using this strategy, we computed pairwise correlation scores between all 25,000 signatures, resulting in over 625 million pairwise scores.

### Meta-analysis to compute signature-ontology correlations

Currently, our system contains tens of thousands of datasets representing diverse types of biological conditions. With the development of new sequencing technologies we anticipate hundreds of thousands of public datasets to be available for the research community in the near future. To systematically interrogate these huge quantities of data, we have to abstract our analysis to the level of biological conditions those datasets represent. Researchers can then look at the connections between their own data and the potentially thousands of individual datasets with matching tissues, diseases, compounds, or genetic perturbations. Ontology-based meta-analysis is designed to accomplish that goal by computing an overall correlation score between a given gene set and an ontology concept (e.g. disease). The meta-analysis algorithm statistically assesses “reproducibility” of significant findings, thus minimizing the chance of random correlations and poor-quality data affecting the final results.

The meta-analysis algorithm aggregated scores for various ontology terms associated with the correlated signatures, weighted by the strength of the correlation score (Figure 4, Methods). This was computed separately for tissue, disease, compound, and genetic perturbation categories. The algorithm considered any available hierarchical relationships of ontology terms and propagated enrichment scores to more general concepts accordingly. Concepts that may have had lower scores than their parent concepts were clustered under the parent. A ranked structure of the most relevant tissues, diseases, and compounds was thus pre-computed for each signature. The advantage of this strategy is that related ontology tags could be associated with each signature semantically. For example, “heart” and “left heart ventricle” can both contribute to the “heart” concept meta-analysis score since “heart” is the parent concept of “heart ventricle”.

As each new signature was added, the meta-analysis computations for existing signatures in the system were also updated. This ongoing process ensured that at a given time the most up-to-date results of the meta-analysis given the current state of the knowledge base were produced. When a query with a given set of genes was performed a total collection of meta-categories, as well as individual signatures were scanned to identify top-ranking normal tissues, diseases, and compounds (Table 1).

### Use Case 1: Comparative Analysis across Normal Tissue and Cell Type Data

#### Analysis of the molecular similarity between brown fat and other normal tissues

A large number of experiments in the public domain provide a great resource for exploring normal tissue biology. It is virtually impossible to create a single comprehensive dataset with gene expression profiles of all tissues and cell types of interest. However, normal tissue datasets from hundreds of independent studies can be scanned using gene sets of interest to identify similarities. This can further our understanding of the relationships between different tissues, different stem cell lineages, as well as mechanisms governing normal and aberrant developmental pathways.

We applied our strategy to investigate molecular properties of brown fat cells and to explore their similarity to a collection of other normal tissues. To achieve this we derived a brown fat tissue gene expression signature from the mouse tissue atlas dataset containing genome-wide gene expression profiles of 61unique tissues and organs (NCBI GEO Accession # GSE1133) [21]. Each gene was ranked according to its fold change relative to the median of all mouse tissues (see Methods). As a result we obtained a tissue signature that consisted of 31,309 probe sets and their associated ranks.

**Figure 5:**
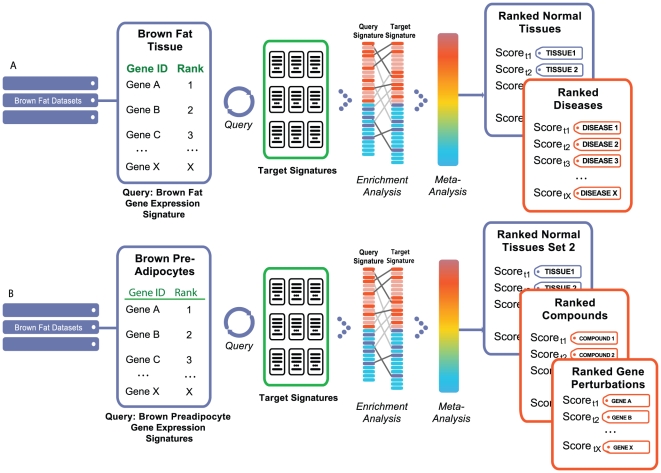
Brown fat meta-analysis. Diagram representing analyses of two different brown fat related signatures: (a) Brown fat tissue signature (relative to all other mouse tissues). (b) Signature of brown preadipocytes vs. white preadipocytes. After computing pairwise scores between query and all target signatures the meta-analysis of pairwise scores and their associated tags (associated disease, tissue, and compound terms) is performed. The final result produces a ranked set of tissues, diseases, and compounds with the most significant association to query signature.

We then used our rank-based enrichment analysis to compute pairwise correlation scores between brown fat and signatures across all studies contained in NextBio (www.nextbio.com, Table 1). A meta-analysis of the pairwise signature correlations then computed the correlation of the brown fat signature with the tags of all target signatures (Figure 5A). The majority of ontology concepts were associated with multiple signatures derived from different studies and organisms. As shown in Table 2, skeletal muscle tissue produced the strongest positive correlation with the brown fat tissue signature. A positive correlation indicates that predominantly the same sets of genes are either up- or down-regulated in the query and target set of signatures (in this case a total of 4 signatures). Skeletal muscle had the top ranked correlation score to brown fat out of 120 total tissue concepts computed from over 2,000 signatures (Table 1). Skeletal muscle data used in the meta-analysis was generated from mouse, rat, and human tissues, providing evidence that the results are consistent across species and platforms. Of interest, these data indicate that brown fat cells are more closely related to muscle than to white adipose tissue, which failed to produce significant correlation with muscle tissue concepts for the same query (Table S2). In a recently published study, Seale *et al.* demonstrated that brown fat cell precursors can turn into muscle cells upon the loss of PRDM16 protein [22]. Other studies have also shown that brown fat and muscle tissue share important molecular characteristics, thus validating our approach [23], [24].

**Table 2:** Brown fat tissue signature query results.

Rank	Normal tissue	Correlation Direction	Correlation Score	# Correlated/ Total Studies	# Total Correlated Signatures
1	Skeletal muscle tissue	+	100	4/4	4/4
2	Tongue	+	93.8	3/3	5/5
3	Epidermis	+	93.3	1/1	3/3
4	Cardiac atrium	+	88.9	2/2	2/2
5	Duodenum	+	85.9	2/2	2/2

The gene expression signature from brown fat tissue was queried against all studies corresponding to normal normal tissues from different microarray platforms and organisms. The results for top five tissues with the biggest positive correlation to brown fat signature are shown. Additional results are shown in Tabs S2 of the Supporting Information section.

### Analysis of a brown preadipocyte signature

Seale *et al.* identified new progenitor cells that gave rise to brown fat and muscle cells but not to white fat cells [22]. Currently, however, there is no gene expression data available for these new precursor cells. Given that, we decided to investigate the molecular properties of another brown fat cell precursor and its molecular similarity with normal tissues and cell types. We derived a brown preadipocytes signature consisting of 2,302 probesets (mouse MG_U74Av2 Affymetrix chip) by comparing gene expression of brown to white preadipocytes (GEO Accession # GSE7032, Table S4) [23]. Using this signature, we performed a correlation analysis across all normal tissues and cell types in NextBio (Figure 5B), and found that muscle stem cells were among the top five concepts with the strongest positive correlation scores to the brown preadipocytes signature (Table 3). This result further contrasts the differences between brown and white fat cells and demonstrates the association of muscle to brown fat. We continued our analysis by looking for patterns as brown and white adipocytes undergo differentiation, and we continued to observe a pattern of positive correlation between brown adipocyte and muscle cell precursors. In accordance with results in Seale *et al.*, our meta-analysis approach suggests the possibility of the existence of a common precursor cell for these two cell types [22].

**Table 3:** Brown versus white preadipocytes signature correlation with normal tissues and cell types.

Normal tissue	Correlation Direction	Correlation Score	# Correlated/ Total Studies	# Total Correlated Signatures
Embryonic tissue	+	89.0	7/7	17/17
Muscle stem cell	+	88.7	1/1	3/3
Hair follicle matrix	+	87.1	2/2	3/3
T-helper type 1 cells	+	82.2	1/1	1/1
Embryonic Stem cells	+	82.1	15/18	26/32

Query results for gene expression signature differentiating brown and white preadipocytes across normal tissue signatures. Positive correlation scores for the top four tissues whose expression signatures correlate with brown vs. white preadipocytes signature (for additional results see Table S5, Supporting Information section).

### Use Case 2: Comparative Analysis across Disease Related Data

#### Analysis of brown fat tissue and correlation with disease signatures

Using public data, we generated thousands of signatures representing 700 distinct disease states. This large collection of disease profiles provides a rich contextual framework with which to explore gene sets of interest. Analysis of tissue- and cell type-specific gene sets against these disease-state profiles can unveil abnormal cell- and tissue-specific programs that are involved in disease development. Analysis of gene sets derived from specific patient cohorts against specific disease signatures can classify a disease more precisely and help drive patient stratification and trial selection [25], [26].

As an example, we investigated the relationship of brown fat tissue to all disease tissue signatures. For the purposes of this study we focused on disease concepts that had a negative correlation to brown fat signature. Among the top ranking disease states, we found obesity, quadriplegia, aging, Duchenne muscular dystrophy, and myocardial infarction (Table 4). The negative correlation to obesity provided a positive control, as brown fat functions in energy expenditure and is associated with resistance to obesity in diverse mouse strains [27]. Furthermore, the proportion of white to brown fat is significantly increased in obese versus normal subjects [28].

**Table 4:** Correlation between brown fat and muscle tissue signatures with diseases.

A. Query: Brown Fat	Rank	Disease	Correlation Direction	Correlation Score	# Correlated/ Total Studies	# Total Correlated Signatures
Brown fat	1	Obesity	-	100	8/14	27/46
Brown fat	2	Quadriplegia	-	78.2	1/1	1/1
Brown fat	3	Aging	-	73.9	27/30	36/48
Brown fat	4	Duchenne Muscular Dystrophy (DMD)	-	86.9	7/8	14/16
Brown fat	5	Myocardial infarction	-	84.1	5/5	25/26

B. Query: Skeletal Muscle						
Skeletal muscle	1	Quadriplegia	-	100	1/1	1/1
Skeletal muscle	2	Duchenne Muscular Dystrophy (DMD)	-	99.9	7/8	14/16
Skeletal muscle	3	Oral cancer	-	89.5	2/2	3/3
Skeletal muscle	4	Nerve injury	-	78.1	4/4	10/14
Skeletal muscle	5	Myocardial infarction	-	77.2	6/6	24/27

Brown fat and muscle normal tissue signatures queried against all disease-related signatures in different studies and organisms. Top diseases with negatively correlated genes to brown fat and muscle are shown (for additional results see Table S3, Supporting Information section).

The strong negative correlation of the brown fat signature with aging (Table 4) may be partly explained by the potential age-related suppression of pathways leading to brown fat cell production. This is supported by the fact that that brown tissue deposits are more abundant in fetuses and newborns, but are less prominent in adults [29]. The strong negative correlation with quadriplegia, Duchenne muscular dystrophy, and myocardial infarction is consistent with our earlier findings of molecular similarities between brown fat and muscle tissues. Also, normal tissue-specific gene expression is suppressed in the atrophied muscles associated with these disease phenotypes [30].

### Use Case 3: Comparative Analysis across Chemical Perturbations Data

#### Brown preadipocytes differentiation signature positively correlates with reversine

A comparative analysis of gene signatures derived from a large collection of compound treatment experiments can identify those compounds with similar biological properties, pinpoint treatments with toxic side-effects, and discover novel indications for existing compounds [11]. Furthermore, by exploring a large collection of compound signatures, investigators can identify chemical perturbations that can activate or deactivate cell type-specific differentiation programs and use them as additional tools in future experiments.

To explore compounds that may affect differentiation of brown preadipocytes, we first derived a differentiation signature of 2,000 probe sets by comparing mature brown adipocytes to brown preadipocytes (Table S6) [23]. We then queried this signature against all compound-related data. The strongest positive correlation discovered was with the signature of the small molecule reversine (Table 5). Interestingly, Kim *et al.* demonstrated that reversine stimulates adipocyte differentiation in 3T3-L1 cells [31]. There is also a strong positive correlation between the signatures of mature brown fat and reversine (Table S7) [32]. This suggests that reversine may be a useful compound in future studies of brown fat and may act to stimulate brown preadipocytes differentiation into mature cells.

**Table 5:** Brown mature adipocytes signature correlation with compounds.

Compound	Correlation Direction	Correlation Score	# Correlated/Total Studies	# Total Correlated Signatures
Reversine	+	100	1/1	1/1
Dasatinib	+	79.8	1/1	9/9
Matrigel	+	79.6	7/8	24/26
Gentamicin	+	79.5	3/5	12/16

Top five query results for gene expression signature comparing mature brown adipocytes to differentiating brown preadipocytes across all signatures tagged with “Compounds” category (for additional results see Table S6, Supporting Information section).

### Use Case 4: Comparative Analysis across Genetic Perturbations Data

#### Analysis of brown versus white preadipocytes signatures

Genetic perturbation experiments represent animal or cell line models in which a gene was deleted, modified, or silenced using transcript-specific siRNAs. Identifying genes whose perturbation causes similar gene expression changes as found in the target condition might help reveal common, key mechanisms involved in the regulation of processes leading to normal and disease development.

To identify genetic perturbations that resulted in altered gene expression patterns similar to the brown preadipocyte signature, we again used the 2,302 probe set derived by comparing the gene expression profiles of brown and white preadipocytes (Table S4) [23]. A query against all genetic perturbation experiments in NextBio (1,235 datasets containing 6,170 gene signatures for a total of 135 perturbed gene products) revealed that perturbations of *SNF5* correlated most positively and those of *MYC* correlated most negatively (Table 6). Positive correlation between brown vs. white preadipocytes signature and *SNF5* perturbation implies that ablation of *SNF5* function induces gene expression changes that are positively correlated with white preadipocytes. Current literature supports the notion that the *SNF5* gene positively regulates adipocyte differentiation during adipogenesis [33]. Our results suggest that *SNF5* expression may direct cell fate towards white preadipocyte differentiation. The negative correlation with *MYC* gene perturbations indicates that *MYC* regulated pathways may positively regulate brown adipocyte differentiation as compared to white adipocytes. A number of reports suggest that overexpression of *MYC* suppresses adipogenesis and that its deletion can stimulate accumulation of white fat in pancreas [34], [35]. Overall, we find that our system allows a deeper look at the regulatory networks involved in regulating brown and white preadipocyte differentiation into mature cell types.

**Table 6:** Brown versus white preadipocytes signature correlation with genetic perturbations.

Perturbed Gene	Correlation Direction	Correlation Score	# Correlated/Total Studies	# Total Correlated Signatures
SMARCB1 (SNF5)	+	100	1/1	1/1
Tcrb	+	96	1/1	10/11
MYC	−	95	21/23	44/65
MYOD1	−	95	5/5	20/28
RHO	−	95	3/3	8/9

Top five query results for gene expression signature comparing brown to white preadipocytes across all signatures tagged with “Genetic Perturbation” category (for additional results see Table S8, Supporting Information section).

## Discussion

In this study we presented a novel strategy for mining global collections of large-scale biological data using a combination of ranked-based enrichment statistics and ontology-based meta-analysis. We have implemented our approach within the NextBio platform (www.nextbio.com) and demonstrated how researchers can use their own gene sets of interest to perform queries in the context of the globally available public data. In a series of case studies, we showed how this strategy can be used to scan thousands of microarray experiments in the public domain against brown fat-related gene sets to glean insight into adipose tissue biology. These adipose-related gene sets were analyzed within the context of gene expression data from normal and disease tissue comparisons, chemical compound studies, and genetic perturbation experiments. Insights drawn from these case studies were consistent with previously published results and also provided a novel foundation for the formation of new hypotheses.

Two key factors driving the significance of our data-driven *in silico* analysis are the sheer volume of data that we independently correlated and ranked with brown fat-related gene sets (over 4,000 experiments comprising 25,000 signatures) and the replication of observed correlations across multiple independent datasets. Using brown fat-derived gene sets, we demonstrated the strategy of exploring tissue development, cell-type specific expression, and disease etiology. Furthermore, we demonstrated the discovery of compounds and genetic perturbations that could potentially influence gene expression programs involved in adipocyte differentiation. The identification of compounds and genetic perturbations also furthers our understanding of cell type-specific expression and helps in designing new experiments to study white and brown fat biology.

Our strategy also provides a method that addresses, at multiple levels, the data quality concerns that are often raised with respect to publicly available data. First, the data goes through rounds of preprocessing, quality control, and curation. Second, all analysis results are rank-ordered according to enrichment statistics. Finally, the meta-analysis framework ensures that the majority of findings are supported by multiple, independent datasets, which significantly increases the overall confidence of our results.

The brown fat case study represents a hypothesis-generation strategy that can be applied to a variety of biological questions. As the amount of large-scale public data continues to grow, such data-driven *in silico* analyses are becoming increasingly important and provide a complementary methodology to traditional hypothesis-driven research. Similar strategies can also be applied to study the function of genes, pathways, and other biological entities of interest. Additionally, within clinical research settings, it can be used to study common and distinct genomic signatures of different patient cohorts or to identify novel drug indications, among other applications.

The ontology-based meta-analysis strategy presented here enables a higher order view of biological connections within the combined corpus of public and user-generated data. As thousands of new datasets become available, such meta-level analyses can provide a practical way to mine vast quantities of diverse large-scale datasets. Microarray-based gene expression data is the obvious starting point, given the large amount of public data that has become available in the last several years. The next logical step for our strategy would be to extend the current framework to incorporate orthogonal data types generated by proteomics, SNP genotyping, and next-generation sequencing platforms. The combination of orthogonal data will ultimately provide a broader view of biological systems and enable comprehensive *in silico* investigations to take place.

## Methods

### Raw data pre-processing

A majority of studies currently processed within NextBio adhere to certain criteria for inclusion:

- Comprehensive coverage of genes - The platform used should contain over 12,000 probes for human, mouse, or rat studies. For all other organisms, the array should contain at least half the number of probes as there are estimated genes in the genome.
- Presence of a baseline or control group.
- Access to raw or normalized expression values
- Sample annotations provided

To ensure standardization in the processing pipeline, studies were processed from raw data whenever available and from pre-processed data otherwise. For example, for Affymetrix-based studies in which CEL files are available, RMA normalization was applied [36]. Otherwise, expression summary intensities, such as those processed generated by MAS5 (Affymetrix) or dChip were processed [37]. All datasets went through appropriate processing steps that depended on the data type, platform, and experimental design used to generate the data and include:

- Background subtraction, if applicable
- Expression summarization, e.g. using RMA when CEL data is available
- Data transformation (log) and technical replicate averaging and negative value correction
- Normalization – RMA, per-chip median or Lowess where applicable
- Quality control assessment
- Statistical (differential expression) analysis

The vast majority of processed data in the system falls into the category of case-control experimental design analyzed using Welch or standard t-tests, paired or unpaired, as appropriate. Quality assessment methods were employed to review sample-level and dataset-level integrity – these included curator review of pre- and post-normalization boxplots, missing value counts, and p-value histograms (after statistical testing) with FDR analysis to determine whether the number of significantly changing genes is greater than expected by chance.

A p-value significance cutoff of 0.05 (without any multiple testing correction) and a minimum absolute fold-change cutoff of 1.2 (typically the lowest sensitivity threshold of commercial microarray platforms) was used to obtain the final set of signatures of differentially-expressed genes. This double filtering procedure serves to address different aspects of variability in the data [38]–[40]. To address the potential unreliability of the fold-change metric at low intensity levels [41], genes with signals lower than a 20^th^ percentile cutoff in both control and test groups were discarded from the signature.

Expression profiles can vary considerably from study to study and from platform to platform. Different platform technologies can yield different dynamic ranges, distributions of fold-changes, and p-values that reflect the technologies used. To allow inter-study comparability, a non-parametric approach was established so that ranks were assigned to each final gene signature based on the magnitude of fold change. Fold-change, as a ranking metric, had a better concordance across platforms than p-values from statistical tests [42]. Ranks were then further normalized to eliminate any bias due to varying platform sizes.

In the absence of a “gold standard” for processing microarray data, these statistical threshold cutoffs serve to maintain a reasonable and consistent level of data quality across all studies analyzed within NextBio and are commonly adopted in the literature [43]–[45]. The thresholds are intentionally permissive to ensure that signatures contain *all* potentially interesting elements. The potential for introducing noise, i.e., more false positives, is balanced by (a) enforcing the basic quality control metrics described above and (b) incorporating a normalized rank-based scheme that captures the relative importance of each gene in a signature. This key metric of the meta-analysis framework is described below. In summary, this strategy results in the following advantages:

1. The normalized ranking approach enables comparability across data from different studies, platforms, and analysis methods by removing dependence on absolute values of fold-change, minimizing some of the effects of normalization methods used, and accounting for platform effects.
2. During pair-wise comparison of signatures, the *Running Fisher* algorithm (described below) dynamically determines the best cutoffs corresponding to the maximal similarity score by scanning all of the potentially interesting data. Most of the time, low ranking genes do not contribute to the maximal score, thus reducing the dependence on the minor variations of the actual cutoffs used in generating biosets.
3. A meta-analysis identifies genes with consistent signals across several experiments. This rescues potentially interesting gene signatures that might otherwise have fallen below the margin of significance in an analysis based on a single study.

### Cross-platform comparisons

An index of microarray platforms was compiled to aid in the comparison of microarray data. The index provides a standardized mapping of commonly used public- and vendor-specific vendor gene identifiers to reference identifiers such as NCBI Entrez Gene, UniGene, Ensembl, RefSeq, or GenBank accession numbers.

### Cross-species comparisons

To enable seamless comparison across different species, orthologs were identified for each pair of organisms and were grouped into ortholog clusters. Ortholog information was derived from Mouse Genome Informatics (MGI) at Jackson Lab (http://www.informatics.jax.org), HomoloGene at NCBI (http://www.ncbi.nlm.nih.gov), and Ensembl (http://www.ensembl.org). Ortholog clusters were generated as follows: 1) the manually curated pairwise ortholog data among human, mouse, and rat from MGI were retrieved and clustered to form initial ortholog clusters. 2) The homology group data among human, mouse, rat, fly, and worm were analyzed to remove those in conflict with MGI data. The filtered homology group data were then entered into the ortholog clusters. 3) The whole genome pairwise sequence similarity data from Ensembl were processed to identify reciprocal best hits as candidate orthologs for all pairwise organisms among human, mouse, rat, fly, worm, and yeast. The candidate orthologs were prioritized based on the percentage sequence identity and examined against the existing ortholog cluster. Qualified ortholog candidates were then entered into the ortholog cluster.

### Computing pairwise correlation scores between gene signatures

The directional relationship between the two signatures is captured by the sign of the correlation score. The up-regulated genes (b^+^) and the down-regulated genes (b^−^) are separated into directional subsets, and correlation scores are computed for each directional subset from one signature (b1^+^, b1^−^) against each subset from the other signature (b2^+^, b2^−^). A positive sign is given to a subset pair of the same direction (b1^+^b2^+^, b1^−^b2^−^), and a negative sign is given to a subset pair of opposite directions (b1^+^b2^−^, b1^−^b2^+^). The overall correlation score is the sum of directional subset scores and the sign of the sum determines whether the two signatures are positively or negatively correlated (Figure 3). The matching genes between two typical gene signatures are depicted in Figure 2. The directionality and ranks for each gene is shown.

The detailed steps given two gene signature sets (*b1, b2*) are as follows:

First, each gene signature set is rank-ordered according to fold change, p-value or a particular score. If appropriate metrics are not provided, then the gene signature set is unranked. The up-regulated genes and down regulated genes are noted with positive and negative signs to imply directionality, respectively. A directional subset is generated for each direction, such as *b1^+^*, *b1^−^*, *b2^+^*, and *b2* ^−^ (Figure 3). If no directional data are provided, then the gene signature set is not directional and only one subset is formed with the whole signature set, such as *b1^o^*, or *b2^o^*.

Second, all the subset pairs are identified: *b1Di*, *b2Dj*, where *Di* and *Dj* are the available directions (+, −, or o) in *b1* and *b2*, respectively. The Running Fisher algorithm is applied to each subset pair. The top ranking genes in the first subset *b1Di* are collected as a group *G*, and the second subset *b2Dj* is scanned top to bottom in the rank order to identify each rank with a gene matching a member in the group *G*. If the subset is unranked, all the genes in the subset are retrieved at the first scan.

At each matching rank *K*, the scanned portion of the second subset *b2Dj* consists of *N* genes, and the overlap between group *G* and *N* genes is *M*. A Fisher's exact test is performed at rank *K*, to evaluate the statistical significance of observing *M* overlaps between a set of size *G* and a set of size *N*, where the set of size *G* comes from platform *P1* and the set of size *N* comes from platform *P2*, given the sizes of *P1* and *P2* as well as the overlap between *P1* and *P2*.

At the end of the scan, the best p-value is retained, and a multiple hypothesis testing correction factor is applied. The multiple testing factor is the expected number of overlaps between the two subsets of the given sizes, given the two platforms *P1* and *P2*. The negative log of the multiple testing corrected best p-value is a score for the subset pair.

Next, the Running Fisher algorithm is performed in the reverse direction: the top ranking genes in the second subset *b2Dj* are collected as a group *G*, and the first subset *b1Di* is scanned in the rank order. The same procedure in this reverse direction produces another score for the same subset pair. The two scores are averaged to represent the magnitude of the similarity between the two subsets. A positive sign is given to the final subset pair-score if *Di* and *Dj* are the same. A negative sign is given if *Di* and *Dj* are opposite. The score is unsigned if any of *Di* and *Dj* is not directional.

Finally, the overall score is computed by summing up all directional subset pair scores (Figure 3). The sign of the sum determines whether the two signatures are positively or negatively correlated. If one of the two signature sets is directional and the other is not directional, the overall score is represented by the larger of the two subset pair scores, annotated with the contributing direction from the directional signature. If both signatures are not directional, then a single unsigned pair score is calculated between the two biosets.

### Ontology-driven Meta-Analysis

Given a gene signature representing the set of genes of interest from a given experiment, a meta-analysis of the tens of thousands of tagged gene signatures in the NextBio system can be used to determine tissues, diseases, and compounds associated strongly with the query set. Conceptually, the problem is that of ranking ontology terms (concepts) based on how strongly enriched signatures tagged with those concepts are with a set of genes of interest. For a set of genes, ranked or unranked, the gene set enrichment analysis described above is used to identify other strongly associated signatures. Based on the strength of the association, the aggregated scores for their associated semantic concepts were computed.

Meta-analysis scores were computed separately for tissue, disease, and compound categories. Given a query gene signature, a list of contributing signatures was obtained for each category based on two criteria – (1) They have enrichment scores with the query signature above a pre-determined threshold and (2) They pass an initial screening logic that ensures that they are tagged with the appropriate combination of concepts to allow them to contribute to that category.

Based on the list of contributing signatures, along with the associated concepts and enrichment scores, the equation below describes the various factors considered in determining a score for a concept.
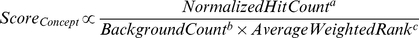



The normalized hit count for a concept is the sum of the ratio of associated score of each signature tagged with that concept to the overall best association score. The background count of a concept is the number of signatures in the NextBio system tagged with that concept. Inclusion of the background count reduces the bias toward popular concepts that have more associated gene signatures than others. Finally, the average weighted rank represents the average rank of a tagged signature relative to all other correlated signatures weighted by the associated normalized score.

Given the dynamic nature of the NextBio system where the distribution of data from various species, platforms, data types, and semantic categories changes on a continuous basis, it is not obvious at the outset what the relative contributions of each of these factors should be toward determining an optimal overall scoring function for determining top ranked concepts. These are determined empirically by optimizing the model described above using gold standard use cases and tuning parameters (a,b,c in the equation above) for each of the factors.

This meta-analysis scoring process results in a ranked list of ontological terms for each tissue, disease, and compound category. It should be noted that some concepts are part of a hierarchical ontological framework. When that was the case, enrichment scores for signatures tagged with specialized concepts are accordingly propagated to more general parent concepts in the hierarchy. After scores for all concepts are computed, children concepts with lower scores than parent concepts are clustered and presented to the user.

### Computing direction of signature-concept correlation

The overall direction of association (positive or negative) between a query signature and a concept refers to the *net* correlation of the query signature with a set of contributing signatures (see previous section) tagged with that concept. Recall that these contributing signatures may have positive or negative pairwise correlation scores with the query signature. A *cumulative* positive and negative score is obtained by aggregating the scores for the positively and negatively associated signatures separately. To minimize the effect of any spuriously strong signature association, the cumulative scores are each *weighted* by the ratio of the respective number of positively or negatively associated signatures to the total number of contributing signatures. Finally, the overall direction is called depending on which *weighted cumulative score* is greater, as long as a minimum difference threshold is met.

## Supporting Information

Table S1 The list of public databases containing raw microarray data available to the public. Only major databases were included in this list.
(0.02 MB XLS)

Table S2 Meta-analysis results for the brown fat tissue signature query against all other public datasets on normal tissue analysis.
(0.02 MB XLS)

Table S3 Meta-analysis results for the white fat tissue signature query against all other public datasets on normal tissue analysis.
(0.38 MB XLS)

Table S4 Brown preadipocytes gene expression signature. The signature was identified by comparing cultured brown preadipocytes to white preadipocytes at day 4.
(0.01 MB DOC)

Table S5 Meta-analysis results for the brown preadipocyte signature query against all other public datasets on normal tissue analysis. Brown preadipocyte signature was determined by comparing gene expression of cultured brown preadipocytes versus white preadipocytes at 4 days.
(0.01 MB XLS)

Table S6 Query results for gene expression signature comparing mature brown adipocytes to differentiating brown preadipocytes across all signatures tagged with “Compounds” ontology category.
(0.01 MB XLS)

Table S7 Reversine gene expression signature. The signature was identified by comparing cultured C2C12 mouse myoblasts treated with reversine to non-treated control myoblasts.
(0.62 MB XLS)

Table S8 Meta-analysis results for the brown preadipocyte signature query against all other public datasets on genetic perturbations analysis. Brown preadipocyte signature was determined by comparing gene expression of cultured brown preadipocytes versus white preadipocytes at 4 days
(0.01 MB XLS)
